# The dynamics of mitochondrial-linked gene expression among tissues and life stages in two contrasting strains of laying hens

**DOI:** 10.1371/journal.pone.0262613

**Published:** 2022-01-13

**Authors:** Clara Dreyling, Martin Hasselmann

**Affiliations:** Department of Livestock Population Genomics, Institute of Animal Science, University of Hohenheim, Stuttgart, Germany; Tokat Gaziosmanpasa Universitesi, TURKEY

## Abstract

The cellular energy metabolism is one of the most conserved processes, as it is present in all living organisms. Mitochondria are providing the eukaryotic cell with energy and thus their genome and gene expression has been of broad interest for a long time. Mitochondrial gene expression changes under different conditions and is regulated by genes encoded in the nucleus of the cell. In this context, little is known about non-model organisms and we provide the first large-scaled gene expression analysis of mitochondrial-linked genes in laying hens. We analysed 28 mitochondrial and nuclear genes in 100 individuals in the context of five life-stages and strain differences among five tissues. Our study showed that mitochondrial gene expression increases during the productive life span, and reacts tissue and strain specific. In addition, the strains react different to potential increased oxidative stress, resulting from the increase in mitochondrial gene expression. The results suggest that the cellular energy metabolism as part of a complex regulatory system is strongly affected by the productive life span in laying hens and thus partly comparable to model organisms. This study provides a starting point for further analyses in this field on non-model organisms, especially in laying-hens.

## Introduction

Understanding the impact of the genetic background and developmental processes on gene expression are of broad general interest to understand organismic function. Through the process of oxidative phosphorylation (OXPHOS), mitochondria are generating 90% of the cellular energy [1]. In addition, these organelles are involved in the maintenance and regulation of intracellular energy metabolism and signalling [2], apoptosis [3, 4] and have major importance in cell cycle regulation [5] and ageing processes [6]. Mitochondria received particular attention in relation to ageing as a modulator of both, the production of damaging reactive oxygen species (ROS) that can cause cellular damage [1] and the synthesis of energy in form of adenosine triphosphate (ATP) via electron transfer mediated by nicotinamide adenine dinucleotide + hydrogen (NADH) [7]. The nuclear encoded Superoxide dismutase (*SOD2)* is converting superoxide anions (O_2_^●-^), (which are the most produced ROS during OXPHOS [8]) to hydrogen peroxide [6, 9]. An increase in the production of ROS in the mitochondrion is linked to the process of ageing in many species [10] and the increase of *SOD2*-expression protects the mitochondrion from damage, which would otherwise lead to the death of the cell (Santos et al., 2018 [10], Yin et al., 2018 [11] and cited references within).

Evidence of diminished performance of mitochondria during the course of life span have been frequently found [12, 13]. To maintain their functionality and their ability to react to external influences, the mitochondrial gene expression is part of a network of transcription factors and other gene products from the nuclear genome [14, 15], supporting the view of an intimate interaction of mitochondria with nuclear genomes.

Within this complex framework of interaction, mitochondria play a crucial role as powerhouse of the cell that have been studied in a variety of model organisms such as in fruit flies (*Drosophila*) and mice [16, 17] but in a much smaller proportion of livestock such as cattle or pigs [18, 19].

In our experimental setup, we use two different strains of laying hens, with the benefit of having a highly bred species for the specific purpose of egg laying, but represented in two genetically distinct lineages at the same time [20]. Since the focus and thus the need for energy and metabolites changes during the hens’ life span, we sampled at five different points, covering growth and egg laying as well the shift between these two phases, and the changes during the laying phase towards its end. Related to this, it is an utmost importance to gain a better understanding of the utilization of phosphorus (P) and calcium (Ca) and the metabolism of *myo*-inositol (MI) during life span within the animals [21, 22]. Since the mitochondrial process of OXPHOS is directly linked to the availability of P [23], and the energy metabolism is closely linked to the animals’ fitness, mitochondrial gene expression is of highest interest in this framework. Furthermore, we included nuclear encoded subunits of OXPHOS complexes such as UQCRC1, which is a component of the ubiquinol-cytochrome c oxidoreductase and thus part of the mitochondrial electron transport chain [24], and other nuclear genes such as e.g. AMP-activated protein kinase (AMPK), which is linked to the regulation of mitochondrial biogenesis and the energy metabolism [25]. AMPK acts as an activator of PGC1α in case of energy deficit, to increase mitochondrial gene expression [26]. In addition, its activation inhibits MTOR [27], which in addition regulates the expression of PGC1α and other transcription factors linked to mitochondria [28] and a decrease in its expression has been linked to an extension of life span in *Drosophila* [29] and yeast [30] as a response to nutrients. The reactions of PGC1α, subunits of AMPK and MTOR have also been described in broilers in the context of feed efficiency and muscle growth [31]. To cover not only the mitochondrial gene expression itself, but also the complex regulatory network, these genes were included in the study as well.

We follow three main hypotheses:

1. Due to the different (mitochondrial) genetic background [20] and different measured performance [21] of the two strains, differences in gene expression are expected as well.
2. Due to the observation in other species [12, 13] and the metabolic changes during the observed time period a change or decrease in mitochondrial gene expression with the ongoing productive lifespan is expected.
3. These changes include a visible change when the hen’s focus shifts from growth to egg laying (period 2 to 3) and after the peak of egg laying towards the end (periods 4 and 5).

In this study, we explore the gene expression differences and changes during life span of two strains of laying hens and provide insight into the genetic machinery linked to the complex energy metabolism of a domesticated animal. Our study benefits from the known genetic background and low mitochondrial diversity of the two strains [20], which provides a robust data base for the analysis of gene expression differences between groups.

## Material and methods

### Animals and experimental setup

The animal experiments were performed at the Agricultural Experiment Station of the University of Hohenheim, Germany. They were approved by the Regierungspräsidium Tübingen, Germany (Project no. HOH50/17TE) in accordance with the German Animal Welfare Legislation.

We used 100 laying hens: 50 brown (Lohmann brown classic) and 50 white (Lohmann LSL classic) white leghorn hybrids contributed by Lohmann Tierzucht (Cuxhaven, Germany). The hens originated from an experiment addressing the utilization of P and Ca in different periods of the hens’ life [21]. The experimental setup is described in detail in Sommerfeld et al., 2020 [21] and will only be outlined briefly in the following.

The hens were reared together under standard conditions, with diets according to the requirements of each period, based on soy and corn meal with no difference to the recommendations as described in Sommerfeld et al., 2020 [21]. Ten father lines per strain were selected based on the average bodyweight of the female offspring prior to the start of the first experimental phase. After 8, 14, 22, 28, and 58 weeks ten hens per strain were selected and placed into metabolism units, to monitor feed intake and collect excreta on an individual basis. The hens were weighed at the beginning and end of this period, to calculate changes in body weight. After the end of this period (10, 16, 24, 30, and 60 weeks) the animals were slaughtered at the Agricultural Experiment Station of the University of Hohenheim [21].

### Samples and RNA extraction

We used fives tissues from each individual: breast muscle, duodenum, ileum, liver and ovary. Samples were directly taken after slaughtering when the hens were 10, 16, 24, 30 and 60 weeks old (denoted in the following as period 1 to 5, respectively) as described by Sommerfeld et al., 2020 [21] and were immediately placed on dry ice. The samples were stored at -80°C until the extraction of RNA.

RNA was extracted from 25mg tissue using TRIzol Reagent (Thermo Fisher scientific Inc., Massachusetts, USA) according to the manufacturers’ instructions. The samples were homogenized using steel beads at 5.5m/s for 40 seconds on a FastPrep24 (MP Biomedicals, Thermo Fisher scientific Inc., Massachusetts, USA) and a centrifugation step was included afterwards, as recommended for samples with high fat content. Samples were dissolved in nuclease-free water and RNA concentration and quality was measured using a NanoDrop 2000/2000c Spectrophotometer (Thermo Fisher scientific Inc., Massachusetts, USA). In addition to the 260/280 and 260/230 ratios provided by NanoDrop for all samples the integrity of the extracted RNA was checked via gel-electrophoresis [32] and on a Qubit 4 (Thermo Fisher scientific Inc., Massachusetts, USA) using the Qubit RNA IQ Assay Kit (Thermo Fisher scientific Inc., Massachusetts, USA) of a random but representative subset (including all tissues, strains an periods as well as different concentrations and 260/230 ratios). The samples were stored at -80°C until further processing.

### Real time PCR

We selected 33 genes including all mitochondrial encoded genes (13), nuclear encoded genes (20), which includes genes important for life span and mitochondrial biogenesis. During the course of PCR evaluation and quality checks, six genes (two mitochondrial and four nuclear encoded ones) needed to be excluded due to suboptimal performance or due to low number of successful reactions (the final subset of genes can be found in Table A in S1 File). A list of the final genes and their abbreviations used in this work can be found in Table 1. Thus, for all subsequent analyses we remain with a set of 27 gene assays. Additionally, we included three potential nuclear encoded reference genes: Actin beta (*ACTB*), Peptidyl-prolyl cis-trans isomerase A (*PPIA*) and Glycerinaldehyd-3-phosphat-Dehydrogenase (*GAPDH*) that have been used in previous studies for this purpose [33, 34].

**Table 1:** Genes used in this study with abbreviations and genome in which they are encoded.

Abbreviation	Genome	Gene
*ACTB*	Nuclear	Actin beta
*ATP6, ATP8, ATP5F1*	Mitochondrial Nuclear	ATP-synthase F_0_ subunits
*COX1, COX2, COX3, COXC6, COX5A*	Mitochondrial Nuclear	Cytochrome oxidase subunits
*CytB*	Mitochondrial	Cytochrome b
*GAPDH*	Nuclear	Glycerinaldehyd-3-phosphat-Dehydrogenase
*IGF-1α*	Nuclear	Insulin-like growth factor 1α
*MTOR*	Nuclear	mechanistic target of rapamycin
*ND1, ND4, ND4L, ND5, ND6, NDUFB6*	Mitochondrial Nuclear	NADH:ubiquinone oxidoreductase subunits
*PGC1α*	Nuclear	Peroxisome proliferator-activated receptor gamma coactivator 1-α
*PPIA*	Nuclear	Peptidyl-prolyl cis-trans isomerase A
*AMPK (PRKAA1, PRKAA2, PRKAB2, PRKAG2)*	Nuclear	AMP-activated protein kinase and its α1, α2, β2 and γ2 subunits
*SDHA. SDHB*	Nuclear	Succinate dehydrogenase complex subunits
*SOD2*	Nuclear	Superoxide dismutase
*UQCRC1, UQCRC2*	Nuclear	Cytochrome b-c1 complex subunits 1 and 2

#### Assay design and evaluation

All primers were designed using Primer3 [35] based on reference sequences from NCBI except the primer-pair for *GAPDH* [34] (Table A in S1 File). Prior to real time PCR all primers were tested using standard PCR with the same conditions as used for the final analysis (Table B in S1 File). DreamTaq Green (Thermo Fisher Scientific) was used according to the manufacturers manual, and the resulting fragments were visualized by standard agarose gel-electrophoresis and sequenced bidirectional using Sanger technique (performed by Microsynth AG (Balgach, Switzerland)) to test for specificity.

All real time PCR analyses were performed on a Biomark HD system (Fluidigm Corporation, San Francisco, USA), following the protocols of the supplier for gene expression analysis. The evaluation of the performance of the assays was done on FlexSix GE integrated fluidic circuits (IFCs) in duplicates per assay. A 12-step 10-fold dilution series (starting with a concentration of 16.6ng/μl) of the previously sequenced specific PCR product was used to determine limits of detection, linear dynamic detection range, variation at detection limit, PCR efficiency and melting curves of the products (via standard curve as described in Bustin et al., 2009 [36]). The PCR cycling conditions were the same as for the final experiments and can be found in supplementary Table C in S2 File. The efficiency was above 90% for 27 primers and above 80% for four of our primer pairs (Table A in S1 File). Melting and standard curves can be found in the S2 File.

#### Final qPCR runs

The analysis was performed on six 96.96 IFCs using the Delta Gene Assays protocol with the manufacturers standard protocol for fast PCR and melting curve as described in supplementary Table C in S1 File. Remaining DNA was digested using DNAse I (Thermo Fisher scientific Inc., Massachusetts, USA) using 2μg RNA extract in each reaction. For reverse transcription 1μl (= 166.67ng) of this RNA was used in each reaction using the Fluidigm Reverse Transcription Master Mix containing a mixture of poly-T and random oligonucleotides. Pre-amplification with the Fluidigm Preamp Master Mix was performed with 1.25μl (~41.6ng) cDNA for 10 cycles using pooled primers that were the same as used for the final qPCR runs. A multiplex control was performed including five samples of different tissues, strains, period and RNA qualities with and without the pre-amplification step. After Exonuclease I digest of the primers the samples were diluted five-fold. In each well of the IFC 2.25μl of the diluted Exo I digested sample were added, resulting in 3.015nl in each reaction chamber. Negative controls were included throughout all preparation steps and on the final qPCR runs as well to test for contamination of the primers and reagents. Additionally, an internal control was used on each chip, to detect potential intra-run variance. All qPCRs were performed in duplicates and the samples were placed randomly on the chips, only grouped by individual to avoid any bias of sample arrangement.

### Data preparation

#### Quality controls

For data evaluation and quality controls the Fluidigm Real-Time PCR analysis software (version 4.5.2) was used. Only Cq-values from reactions with logarithmic increase of fluorescence and specific melting points were used for the following analyses. After the automatic quality check of the software, the results were evaluated by eye and revised manually if necessary. The quality threshold was set to 0.65 and the peak ratio threshold to 0.8.

The results of the internal control were used to detect possible variation due to technical issues.

#### Reference gene evaluation

To evaluate if the three candidate genes for normalization (*ACTB*, *PPIA* and *GAPDH*) were constant under our experimental conditions, Normfinder [37] was used. As input we used the quality checked data of one brown and one white individual per period and included all five tissues to cover all factors of the experiment. Normalization was tested for tissue type and period.

#### Calculating relative gene expression

Means of duplicates were calculated of all samples with two successful runs. For samples that only had one successful duplicate this run was used. Gene expression relative to the reference genes was calculated using the Pfaffl-method [38] as optimized for multiple reference genes [39, 40]:

$$rel.\;gene\;expression=\frac{{RQ}_{GOI}}{geomean[{RQ}_{refs}]}$$

Where RQ = E^Δct^ and E = (primer efficiency [%]/100) + 1.

Δct was calculated as the difference between the average cycle threshold (ct) of the internal control to the ct of the corresponding sample.

### Statistical analyses

#### Hierarchical clustering

Two-way hierarchical clustering analyses were performed in JMP Pro (Version 15, SAS 199 Institute Inc., Cary, NC, 1989–2019) using Ward’s minimum variance method [41] based on relative gene expression values of all 29 genes. The data were not standardized and only samples without missing data were included. To estimate the best number of clusters the cubic clustering criterion [42] was used as implemented in the program. The process was done for both strains together, and for each strain separate.

#### Analysis of individual genes

A linear mixed effects model was implemented and used for all genes:

Y~strain+period+tissue+tissue*strain+tissue*period+strain*period+tissue*strain*period+individual+father+ε

Where Y is relative gene expression, ε is the residual error, strain, period and tissue are fixed effects, with individual and father as random effects. All modelling was performed in R (R Core Team 2019, Version 3.6.1) using the *lmerTest* package [43]. A three factorial analysis of variance (anova) was performed to evaluate the influence of fixed effects and pairwise tukey *posthoc* tests (package *emmeans* [44]) were performed to detect differences between strain, period and tissues in various combinations based on the estimated marginal means (emmeans) derived from the model. The fulfilling of normal distribution and the homogeneity of variance were evaluated using QQ and residual plots. Outliers were removed for each dataset using the interquartile range prior to the statistical analysis except for *SDHA*, *MTOR*, *PRKAG2* and *GAPDH* where a removal would lead to a strong bias of the analyses.

Means and standard derivations were calculated over the emmeans of all genes to compare the gene expressions between tissues and periods in general. To avoid performing statistical tests on the results of other statistical tests and generating hardly interpretable results, the means were used only for visualization.

## Results

### Sample quality

RNA concentrations ranged from 340ng/μl to 10556.5ng/μl. The results of the NanoDrop and Qubit measurements can be found in the (S3 File). The 260/280 ratios for all samples were close to the optimum of 2, while the ratios were lower for older individuals from period 5 in comparison with the other periods. The same observation was made for the 260/230 ratio where the values decreased with increasing age of the animals and were more diverse in general. Since all samples were treated equally, the differences might result from the age of the individuals themselves, since age related changes in tissues are described for connective tissues [45] and in context of lipofuscin [46]. During the downstream processes no dependencies between lower ratios and qPCR success were observed. The IQ values were above 8 for all except one tested sample independent of the concentration, 260/230 or 260/280 ratios. The high IQ value indicate a proportion from more than 80% of large or/and structured RNA (mRNA, tRNA, rRNA). Samples with high concentrations were diluted previously to the DNAse treatment, which also dilutes the concentration of potential contaminants such as phenol or carbohydrates. Due to the dilutions, no pattern of low performance in combination with low 260/230 ratios or RNA degradation was observed; thus all samples were used for the statistical analysis.

### Final dataset

We identified gene expression of *PPIA* and *ACTB* to be most consistent for using them as reference genes for the normalization of our dataset. The expression of *GAPDH* showed high variation between tissues, and was thus included in our study as nuclear candidate gene instead as reference gene.

After the removal of low-quality Ct values, we received a dataset of 12,628 relative gene expression values including 493 from 500 samples and 28 candidate genes. For 252 samples all genes were run successful, for all other samples values for at least one gene were missing. Missing values originated from the previous filtering or failed runs, whereas we observed no dependencies between sample quality, RNA concentration or sample group (tissue, strain, period) and missing values. Due to the stringent filtering criteria applied to the dataset prior to the analysis, and the filtering for outliers of each gene separately during statistical analysis, together with the high number of samples, we decided to analyse the complete dataset. On average 447.5 samples per gene (min. 296 for *ND5* and max. 486 for *CytB*) entered the final analysis. A detailed table including sample numbers per gene can be found in S2 Table.

The calculated ΔCt values ranged from -7.339 (min for *COX1*) to 11.89 (max for *GAPDH*). The calculated ΔCt values can be found in S5 File.

### Hierarchical clustering

**Fig 1:**
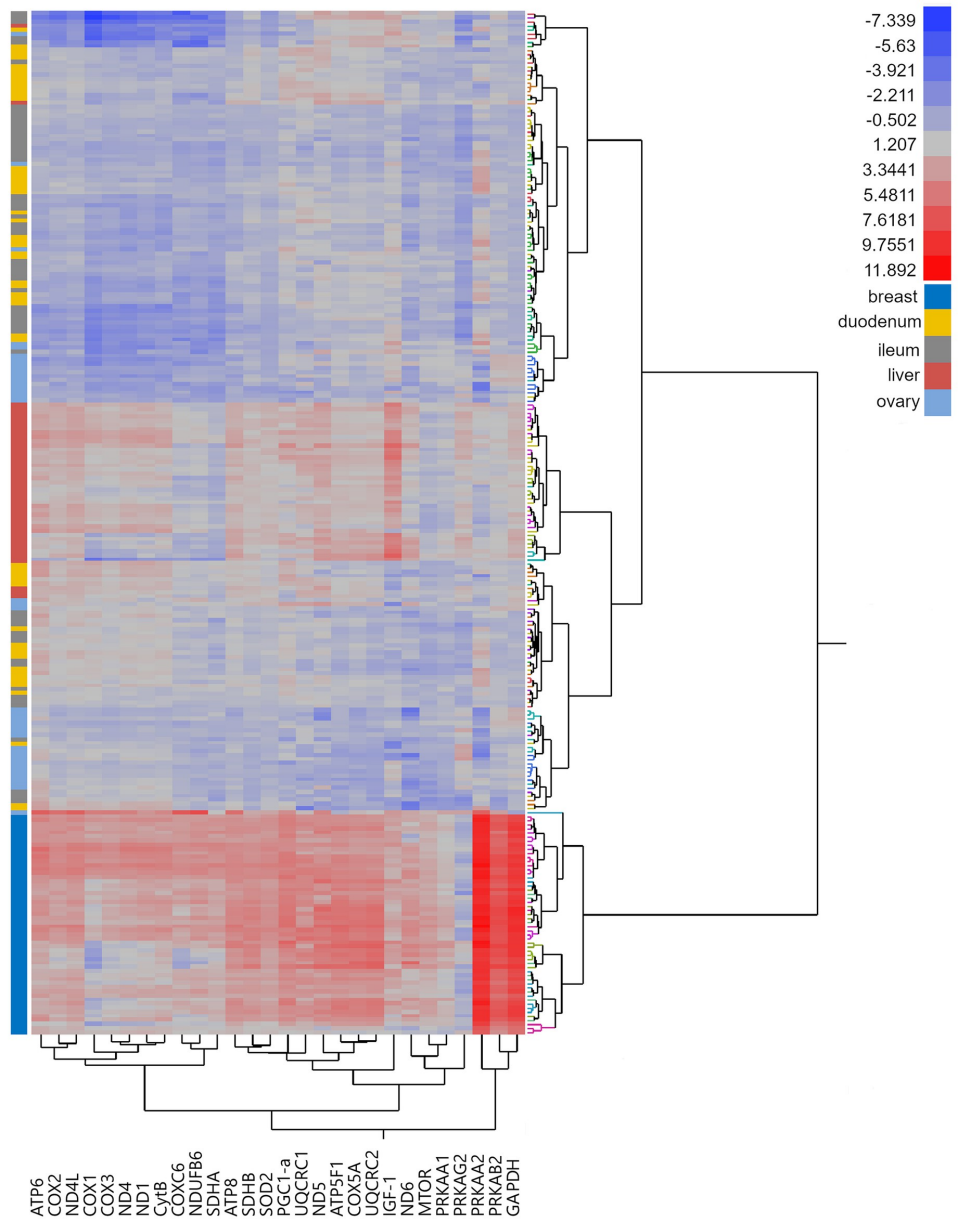
Heat map of two-way hierarchical cluster analysis of the gene expression of 252 samples. 28 Mitochondrial and nuclear genes (Table 1) were used for five tissues obtained from 94 laying hens. Ward’s minimum variance method [41] was used, the number clusters was estimated using the cubic clustering criterion [42]. Colours of branches of the right indicate clusters, coloured bars on the left tissue types.

The hierarchical cluster analysis of the merged dataset was performed on 252 samples, containing 54 breast muscle, 55 duodenum, 60 ileum, 44 liver, and 39 ovary samples, 134 samples from brown, and 118 from white individuals. All five periods were included. The CCC estimated 26 as the best number of clusters. All breast muscle samples formed one coherent cluster, containing no other tissue type (Fig 1). A second coherent cluster was built by 39 liver samples. The other tissue types formed smaller clusters but were more admixed compared to breast muscle and liver. The same observation was made for strain and period: no bigger cluster contained only samples of one strain or period. The clusters containing breast muscle tissue showed higher gene expression values compared to the other tissues, especially in *PRKAA2*, *PRKAB2* and *GAPDH*. The hierarchical cluster analyses on the separated datasets showed the same pattern as described above for both strains but the number of clusters decreased to 14 in brown and 12 in white (S1 Fig). In the white strain, the ovary samples formed a third larger cluster.

### Influence of strain, period and tissue

The linear mixed model showed that all genes were significantly influenced by the tissue, while the influence of the period was affecting 17 and strain only four genes. The detailed results of all analysed genes can be found in S1 Table. The most frequent interaction was between tissue and period (20 genes), followed by strain*tissue*period (7), and the interaction of strain*tissue (4) while strain*period was only influencing the expression of one gene (*NDUFB6*).

### Gene expression differences between tissues

**Fig 2:**
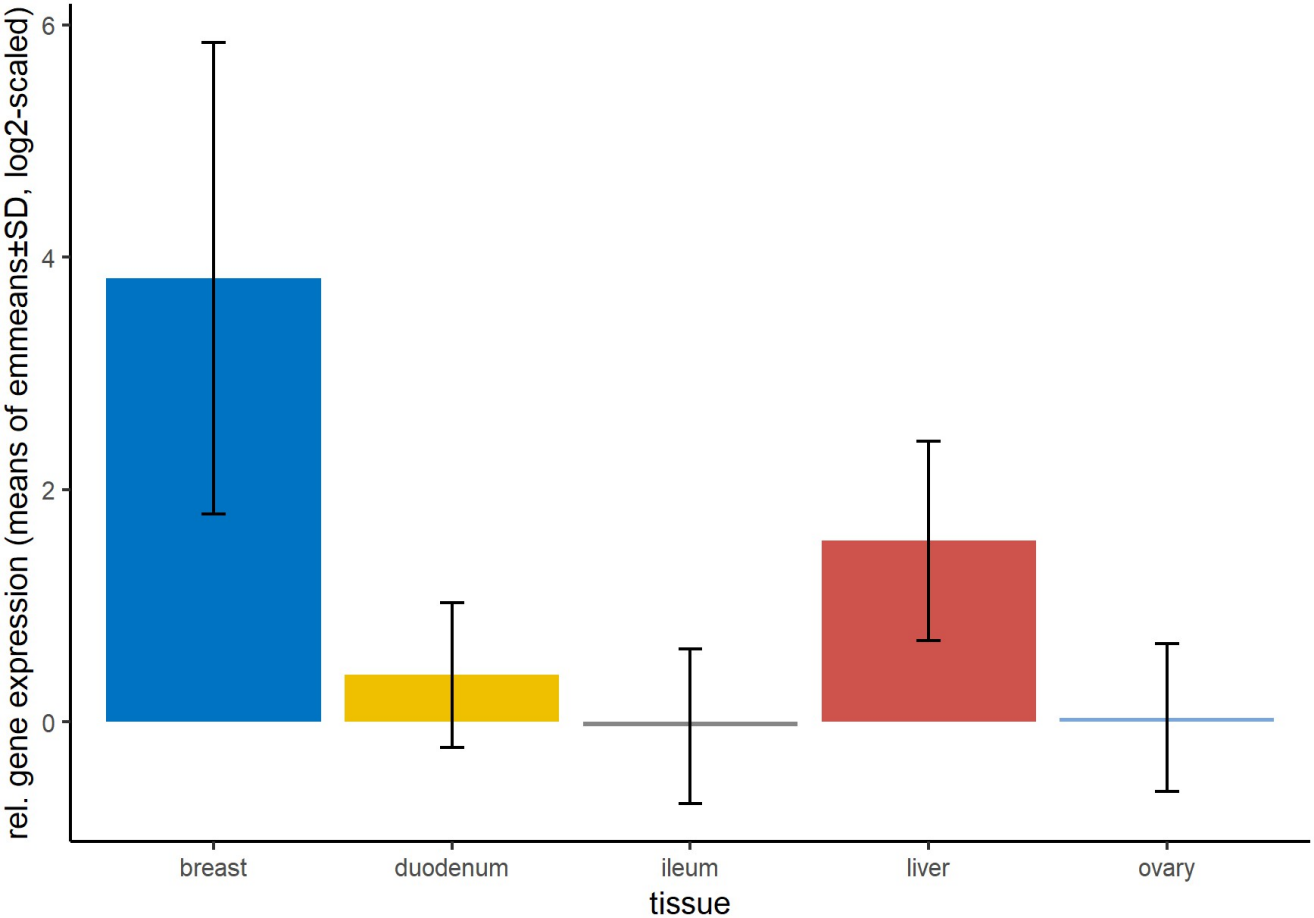
Relative gene expression of all analysed genes per tissue. Shown are means and standard derivation of emmeans over the course of strain and period, calculated using the linear mixed model. Number of samples per group can be found in S2 Table. No statistical test was applied on the shown means of emmeans.

Independent of the period, the gene expression was highest in breast muscle tissue, followed by liver tissue and was lowest in the ileum (Fig 2). Since gene expression differed between periods for many genes in our dataset, the scattering represented by the standard derivation is high but general trends can be observed.

The gene expression was highest in breast muscle tissue for all genes, except for *IGF-1α* and *PRKAG2* where liver tissue had the highest expression. However, the difference between breast muscle and all other tissues was significant for all tested genes (p<0.0001).

### Gene expression differences between periods

**Fig 3:**
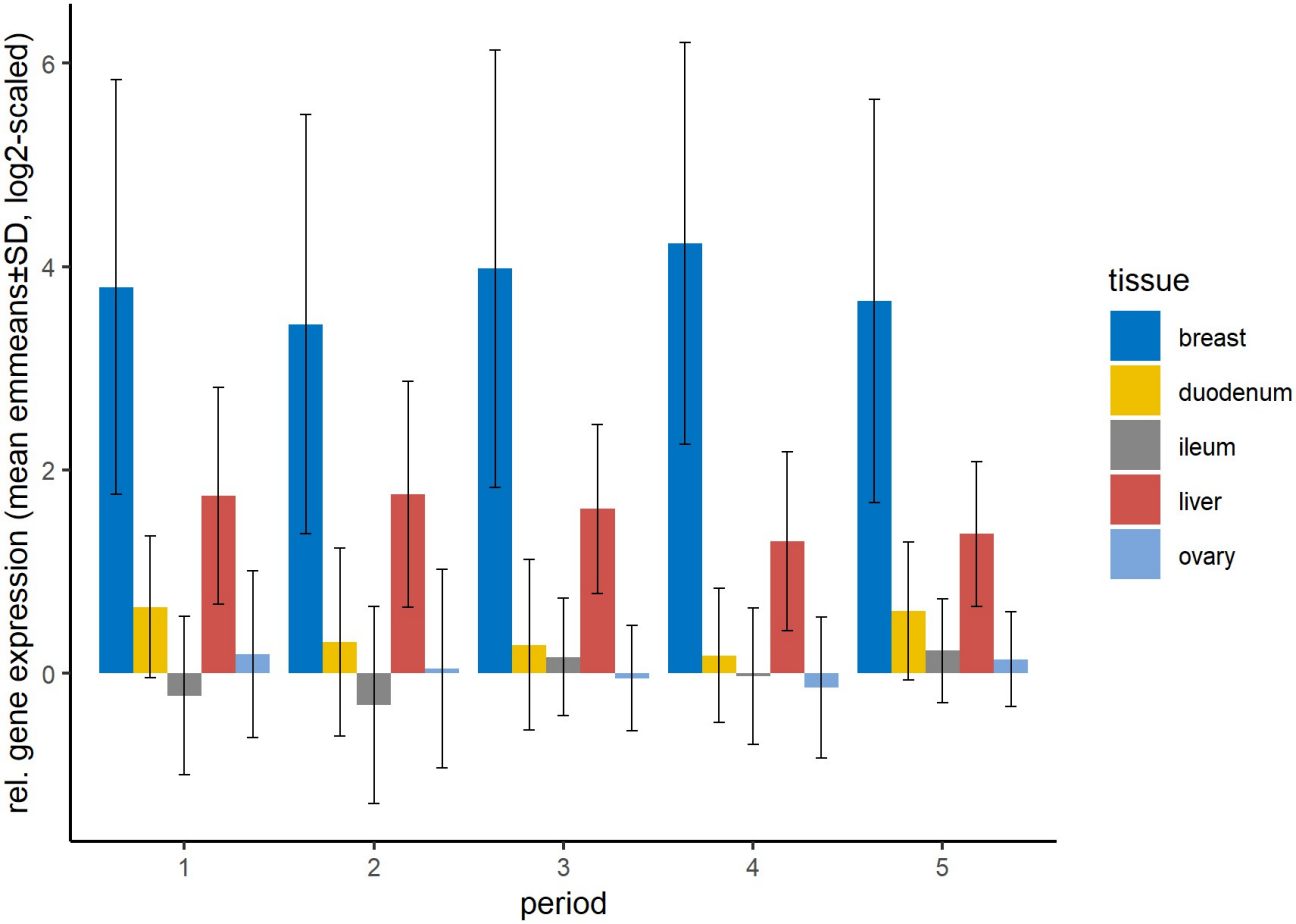
Relative gene expression of all analysed genes per tissue and period. Shown are means of emmeans over the level of strain, estimated by the statistical model with standard derivations. Number of samples per group can be found in S3 Table. No statistical test was applied on the shown means of emmeans.

The period had less influence on gene expression than the tissue, and not all tissues behaved the same way during the periods (Fig 3). In breast muscle tissue the mean gene expression of all genes decreased from period 1 to period 2 followed by an increase and peak in period 4. In liver, the expression was lower in period 4 and 5 compared to the first three periods, in ovary and duodenum the gene expression increased in period 5. The ileal gene expression declined in period 2 and reached a peak in period 3. As described before, the gene expression was highest in breast muscle and liver tissue throughout all periods.

**Fig 4:**
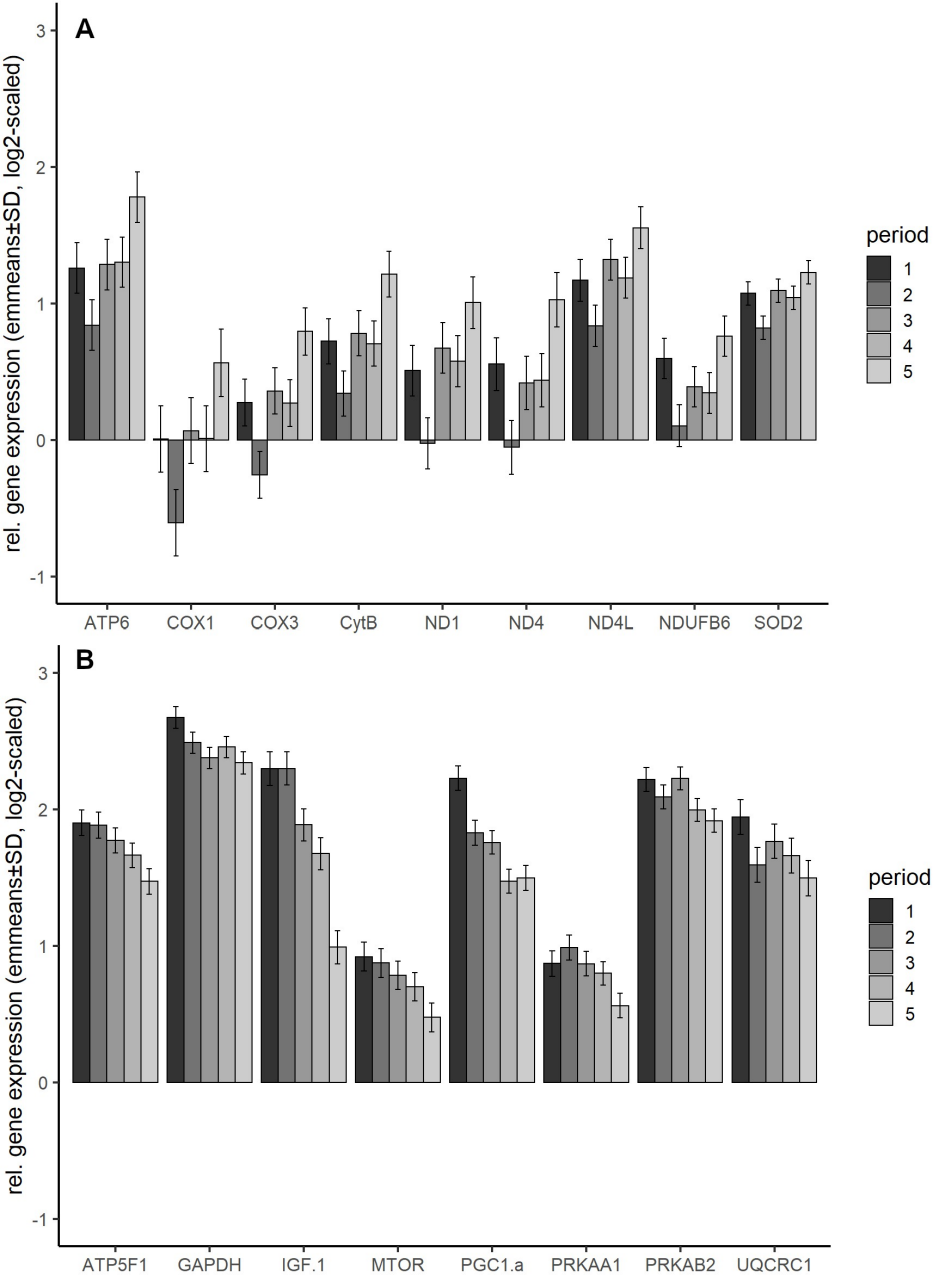
Relative gene expression of genes influenced by period. (A) increasing gene expression (B) decreasing gene expression. Shown are emmeans and standard error, averaged over strain and tissue, calculated using the statistical model. Number of samples per group can be found in S4 Table.

From the 17 genes that were influenced by period, the expression of *ATP5F1*, *GAPDH*, *IGF-1α*, *MTOR*, *PRKAA1*, *PRKAB2*, *UQCRC1*, and *PGC1α* decreased, while the expression of *ATP6*, *COX1*, *COX3*, *ND1*, *ND4*, *ND4L*, *CytB*, *NDUFB6* and *SOD2* and increased with period (Fig 4). In all decreasing genes (except *PRKAA1* and *PRKAB2*), the difference between period 1 and 5 was significant (p values in Table A in S4 File). In *ATP5F1* the expression in period 2 was significantly higher as in period 5 (p = 0.0181) as well as in *PRKAA1* (p = 0.0085). In *PGC1α* the expression in period 1 was significantly lower than in period 4 (p<0.0001). Most significant differences between periods were observed for *IGF-1α*: the expression in period 5 was significantly lower than in all other periods and the expression in period 4 was significantly lower than in the first two periods.

All genes with increasing gene expression showed decreasing gene expression in period 2 and a significantly lower expression compared to period 5. In *COX3* and *ND1* the difference in expression between period 2 and 3 was significant (p = 0.0329 for *COX3* and p = 0.0208 for *ND1*), too. A table with detailed gene expression and p-values can be found in Table B in S4 File.

### Gene expression differences between strains

**Fig 5:**
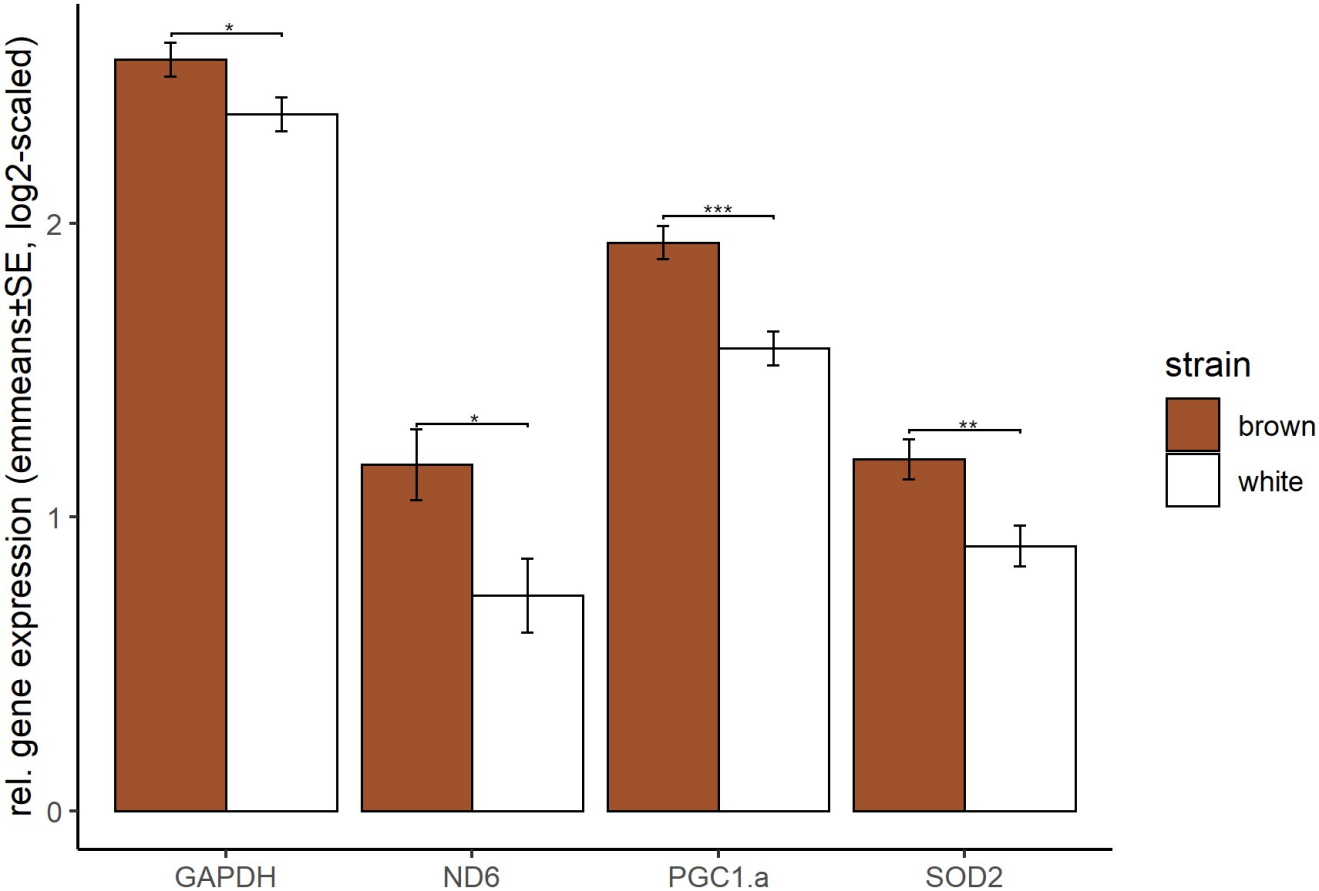
Gene expression differences between the strains. Shown are emmeans and standard errors averaged over the levels of tissue and period for genes with different relative gene expression between both strains. Statistical significance was declared when p < 0.05.

From our variables of interest, strain had the lowest effect on gene expression. Over all periods and tissues only *SOD2*, *GAPDH*, *ND6* and *PGC1α* were differently expressed between the two strains, with significant higher expression in the brown strain (Fig 5). When testing on the period level, the number of differently expressed genes between the strains increased from two or three genes in the first three periods towards five and nine genes in the last two periods. Most differences between the strains were found in breast muscle tissue, not exclusively for genes that were influenced by strain in general.

More in detail, most of the strain differences appeared in PGC1α expression (Table 2): The gene expression was significantly higher in the brown strain in all periods, except for period 2 and was also higher in liver and breast muscle tissue. The expression of *SOD2* was significantly higher in the brown strain in period 4, period 5 and in liver tissue (Table 2). The only gene expression differences with lower gene expression in the brown strain compared to white appeared in *IGF-1α* in period 5 (Table 2).

**Table 2:** Gene expression differences in four genes that are influenced by strain. Shown are emmeans with standard error and p-values, calculated by the statistical model.

	Brown (emmean± SE)	White (emmean± SE)	p
***GAPDH***			
**Period 5**	2.52±0.111	2.16±0.117	0.0273
**Breast muscle**	9.1389±0.113	8.5348±0.119	0.0003
***ND6***			
**Period 1**	1.191±0.247	0.458±0.273	0.0489
**Period 5**	1.127±0.227	0.426±0.242	0.0379
**Ileum**	0.27145±0.189	-0.39683±0.215	0.0214
***PGC1α***			
**Period 1**	2.46±0.126	2±0.129	0.0124
**Period 3**	1.95±0.124	1.56±0.121	0.0275
**Period 4**	1.68±0.126	1.26±0.122	0.0197
**Period 5**	1.69±0.119	1.3±0.135	0.0322
**Breast muscle**	5.16±0.123	4.287±0.121	<0.0001
**Liver**	1.808±0.126	1.251±0.141	0.0036
***SOD2***			
**Period 4**	1.308±0.121	0.774±0.120	0.0024
**Period 5**	1.429±0.119	1.023±0.125	0.0208
**Liver**	1.81813±0.11	1.23718±0.112	0.0003
***IGF-1α***			
**Period 5**	0.707±0.166	1.271±0.178	0.0229

## Discussion

In our experimental setup we were able to analyse a vast number of mitochondrial-linked genes in the context of changes during life span and differences between representative tissue samples in two strains.

### Contrasting gene expression of mitochondrial and nuclear genes during life span

Contrary to our expectation, we observed no decrease in mitochondrial gene expression in the course of our experiment. Instead, the expression of mitochondrial genes that were influenced by period, increased. Interestingly, all the genes followed the same pattern: a decrease in period 2 followed by a constant increase. Manczak et al., 2005 [13] described a similar pattern for genes of the complexes I, III, IV and ATP6 in mice brains: an increase in 12 and 18 months old individuals, followed by a decrease in 24 month old mice (compared to the expression with two month). Even if the time points chosen in the studies differs, the different life expectations of mice and hens suggest, that the observed increase in gene expression might be followed by a decrease later in life, since our study covers the life span that is of agricultural interest, and not the complete potential life span of the birds.

The nuclear encoded *SOD2* gene was also decreasing in the second period and increased in the following periods. *SOD2* protects the cell from oxidative damage deriving from the produced ROS during oxidative phosphorylation, which might explain the increase in gene expression in later periods. This theory is supported by the simultaneously increase of the expression of subunits of complex I and III, which are the main producers of ROS in mitochondria [6]. The observed higher increase of *SOD2* expression in the brown strain, especially in the later periods indicates a different reaction to oxidative stress and might suggest, that the brown strain is better coping with this situation.

The observed increase in mitochondrial gene expression during the productive life span, towards the peak of egg laying (period 4) and towards the end of the laying phase might suggest, that the energy-requirement and related need for ROS detoxification increases with ongoing egg-laying.

Beside the genes that are involved in the process of OXPHOS and ROX detoxification, six genes showed a decrease with ongoing life-stages, which all belong to a complex network: PGC1α, IGF-1α, two subunits of *AMPK* and *MTOR*

PGC1α is a known key regulator of both, the expression of genes involved in the respiration chain and mitochondrial biogenesis [15] and the expression of detoxifying ROS such as SOD2 [47, 48]. The higher expression of both *PGC1α* and *SOD2* in the brown strain in the later periods support our hypothesis that the different strains are reacting differently in the course of their development. Consequently, with an increase of ROS production during life span the expression of enzymes coping with the oxidative stress is needed. On the other hand, *PGC1α* is down regulated in our experimental setup, while the expression of many genes that are affected by the PGC1 family including *SOD2* are up regulated or not affected by the age of the birds.

IGF-1α is another important player within this network, which can be inhibited by AMPK [49] or increase the expression of AMPK [50] under different conditions and work as a nutrient sensitive regulator [6]. However, the regulatory mechanisms between IGF-1, AMPK and PGC1α are poorly understood yet [50]. IGF-1α is an important growth-factor, which is a plausible explanation for the decreasing expression after the first two periods (with significant differences between the first and the last two periods), when the physiology of the hen switches from growth to egg laying.

### Breast and liver tissue represent high levels of gene expression

As expected, we observed a strong influence of tissue on the expression of all genes in our study. However, we were surprised to find the highest gene expression in breast muscle tissue, especially since we are investigating laying hens, which are not bred to primarily gain weight. Interestingly, we observed no increase of gene expression in ovary tissue during the shift from growth to egg laying (period 2 to 3), but an increase towards the end of the laying period (Fig 3).

### Gene expression differences between the strains

We hypothesized to observe differences between the two strains, and also included the father as a random factor, because the individual genetic background might influence gene expression. Interestingly, the strain had the least influence on gene expression as shown by the statistical model. However, we observed that even if both strains are following the same pattern of tissue and period differences, there are differences in some genes. The differences in *SOD2* have already been discussed in the section about contrasting gene expression. In all four genes with significant strain differences independent of period and tissue (*GAPDH*, *ND6*, *PGC1α*, and *SOD2*) the brown strain shows higher expression. The fact, that *GAPDH* is one of these genes supports our initial decision to exclude it as reference gene, additionally to the observed differences between tissues, which have already been shown in human [51]. Interestingly, *PGC1α* showed the most strain differences, while the genes which are regulated by this factor do not differ. However, the fact that two important genes regulating mitochondrial biogenesis (*PGC1α*) and the reduction of oxidative stress (*SOD2*) are significantly higher expressed in the brown strain suggests, that both strains differ in the way they react to changes during the productive life span, especially in the later periods and in highly active tissue such as liver.

An important aspect we expected but did not observe in the broad panel of our data set were differences in the expression of *IGF-1α*. Despite the significant differences in body weight while showing no difference in feed intake observed by Sommerfeld et al., 2020 [21] in the same animals, we only observed strain differences in period 5, where the lighter white strain shows significantly higher expression (Table 2). As a growth factor, *IGF-1α* has been linked to body weight in chicken [52] but seems not to be one of the key players in our experimental setup.

### Gene expression of subunits from the same complex differs

Interestingly, not all subunits of a complex followed the same expression pattern over the different periods. For most complexes, the affected subunits (*COX1* and *COX3*, *ND1*, *ND4*, *ND4L* and *NDUFB6*) followed the same pattern, however, the rest of the subunits of the same complex were not affected by time. The only exception was *CytB* and *UQCRC1*, where the expression of mitochondrial subunit was increasing, while the expression of the nuclear subunit decreased. For the complexes of the respiration chain, studies showed, that the majority of the genes belonging to the same complex seem to be co-expressed and thus, follow the same pattern among different conditions [53]. However, the expression regulation of mitochondrial genes depends on several factors, and expression differences between subunits of complex I have been observed in mice brains [13]. The authors suggested, that the up regulated subunits might be more sensitive to oxidative damage, and thus the organism tries to compensate the resulting loss of mitochondrial function with increased gene expression. In addition, it is known that not all subunits of protein-complexes are regulated in the exact same way [53] and thus, the expression of single subunits can work as a regulatory mechanism of the whole complex [54, 55].

## Conclusion

We performed the first large scale study investigating mitochondrial gene expression in the course of productive life span of laying hens. Our data provided insights into the complexity of this regulatory network by including both, mitochondrial and mitochondrial-linked nuclear genes. In addition, we were able to show, that mitochondrial gene expression is increasing during the productive life span of laying hens, including the ROS detoxifying gene *SOD2*. These findings suggest, that the energy requirements might change during the phase of egg-laying and the organism reacts with an increase in mitochondrial gene expression. The reaction to this increased oxidative stress differs in case of the expression of *SOD2*. The complexity and number of included genes provide a first, initial insight into mitochondrial linked gene expression, whereas for exploring the full expression pattern and underlying regulatory netorks more in detail, transcriptomic analyses are the next logical step as recently shown in Omotoso et al., 2021 [56].

## Supporting information

S1 File Information about primer pairs and cycling conditions. (DOCX)

S2 File Standard and melting curves of all primer pairs included in the study. (XLSX)

S3 File NanoDrop measuremnts of all extracted samples. Samples that were used in the hierarchical cluster analysis and for that all genes run successful are marked.
(XLSX)

S4 File Genes that were influenced by period, with significant period differences. (DOCX)

S5 File Relative gene expression values (log2) calculated using the Pfaffl method and used as input for statistical analyses. (XLSX)

S1 Table Significant influence of strain, period, tissue and all possible interactions on gene expression per gene. p-values from the three-factorial anova obtained from the linear mixed model. Statistical significance was declared when p < 0.05.
(DOCX)

S2 Table Number of samples per gene and tissue after the removal of outliers used to calculate emmeans from the statistical model. (DOCX)

S3 Table Number of samples per gene, tissue and period after the removal of outliers used to calculate emmeans from the statistical model. (DOCX)

S4 Table Number of samples per gene and period after the removal of outliers used to calculate emmeans from the statistical model. Included are only genes influenced by period shown in Fig 4.
(DOCX)

S1 Fig Heat maps of two-way hierarchical cluster analyses of both strains analysed separately. Ward’s minimum variance method [41] was used, the number clusters was estimated using the cubic clustering criterion [42].
(TIF)
