# Supplementary material for: The dynamics of mitochondrial-linked gene expression among tissues and life stages in two contrasting strains of laying hens
Source: PLoS One. 2022 Jan 13;17(1):e0262613. doi: 10.1371/journal.pone.0262613 (PMC8757906; doi:10.1371/journal.pone.0262613)
Supplement: S1 File — (DOCX) [file pone.0262613.s001.docx]

**Table A:** Primer sequences for 30 genes that were used in this study with product size, primer efficiency and accession number of the reference sequence that was used to design the primers. The primer of GAPDH is already published in Hong *et al.* 2012.

| Gene | Forward | Reverse | Product-size [bp] | Efficiency | Accession  Number |  |
| --- | --- | --- | --- | --- | --- | --- |
| *ACTB* | GCTGTGCTGTCCCTGTATGC | TTCTCTCTCGGCTGTGGTGG | 210 | 95.08 | NM_205518 |  |
| *ATP6* | CCGATGGATCAACAACCGCC | AGCTGGGTAGTTGGGGTGAA | 189 | 98.26 | AP003317 |  |
| *ATP5F1* | GCCAGAGAAGGGAGGAGAGG | TGACGCCGGTTTTGGGATAGA | 80 | 106.93 | NC_052557.1 |  |
| *ATP8* | GCCCCAATTAAACCCAAACCCA | TGCAGGGTTGTTTGTTAGAGTGA | 112 | 105.34 | AP003317 |  |
| *COXC6* | CCTTTTGGCCAGGCGGATGA | TTGGGCGGTGCAGACTCAAA | 152 | 96.93 | NM_001198657.2 |  |
| *COX1* | AGCCTTCTAATCCGCGCAGA | AGGAGGGAGGGAGGAGTCAG | 226 | 94.72 | AP003317 |  |
| *COX2* | GACCACGCCCTGATAGTCGC | GGGCAAGCAGGACTAGGACA | 157 | 102.28 | AP003317 |  |
| *COX3* | CCACTACAGCTCGACCACCC | GGTGTTGGGGCTAGGCTTGA | 225 | 107.68 | AP003317 |  |
| *COX5A* | CCACGGGTCACAGGAGTCAG | TGAAATACGTCACCCAGCGG | 56 | 95.17 | NC_052541.1 |  |
| *CytB* | CCCAGCCCCATCCAACATCT | AGGCCTCGTCCGATGTGAAG | 243 | 88.2 | AP003317 |  |
| *GAPDH* | TGCTGCCCAGAACATCATCC | ACGGCAGGTCAGGTCAACAA | 142 | 103.12 | K01458 | |
| *IGF-1α* | TGGCCTGTGTTTGCTTACCTTA | AGCCTCTGTCTCCACATACGA | 107 | 92.61 | JN_593011-18 | |
| *MTOR* | GGCACTGTGTCTATTCTCCAACA | GCAGGTCCTTGGCAGCTTTC | 85 | 93.98 | XM_417614.7 | |
| *ND1* | TCCCCATCCTAATCGCCGTG | GGGAATGGTAGTGGGAGGGG | 250 | 95.42 | AP003317 | |
| *ND4* | CACCCACCCCAACCTACCTG | AGGGTGAGGAAGGGGTAGTGT | 249 | 95.60 | AP003317 | |
| *ND4L* | GTCTCCCCTACACTTCAGCTTCT | GGGCGAATGATGGGGTTTGG | 170 | 91.09 | AP003317 | |
| *ND5* | CCCACCCAAACCAAACACCC | CATAGGCACGTTGTCAGGGC | 229 | 89.91 | AP003317 | |
| *ND6* | TCAACGAGCCCTCCCAGAAC | GGGTTGGTGGTAGCGTCTGT | 236 | 98.57 | AP003317 | |
| *NDUFB6* | CTGATCCCTGCCTGGCTCC | GCCTTCTTTGCTGCAGGACG | 196 | 94.99 | NM_001277525.2 | |
| *PGC1α* | ACAAAAGCCACAAAGACGTCCCT | GCTGCTGTTCCTGTTCTCTGCT | 100 | 100.69 | NC_052535.1 | |
| *PPIA* | TGACTTTACGCGCCACAACG | TCGGTCTTGGCAGTGCAGAT | 165 | 93.55 | NC_052553.1 | |
| *PRKAA1* | GCGGCAGATAAACAGAAGCACG | CGTGTCGCCCAGAATGTAATGC | 66 | 97.63 | NC_052572.1 | |
| *PRKAA2* | TGACGGGGCACAAAGTAGCA | TGAGGGTGCCGGAAGAGTTT | 115 | 96.51 | NC_052539.1 | |
| *PRKAB2* | GAACACCACCAGCGAGCGAG | ACTGGGGTCATCTGTGCTGC | 133 | 89.7 | NC_052539.1 | |
| *PRKAG2* | AGCAGCCCACGTTTCCTCTT | GCCGCTGGTTTTGAGGTAGC | 175 | 105.29 | XM_015281206-9 | |
| *SDHA* | TGTTGTGGGTGCAGGAGGAG | TGGGGCTTGCTCAGTCATGT | 247 | 96.03 | NM_001277398.1 | |
| *SDHB* | TCGAGGAGCTCAGACGGC | CCCCAGGCTTATCAGGATCCC | 83 | 95.03 | NC_052552.1 | |
| *SOD2* | ACACTCTTCCTGACCTGCCTT | CCTTTTGCCAGCGCCTCTTT | 151 | 95.81 | NC_052534.1 | |
| *UQCRC1* | CCCTGCTGCCTCTGACGA | GTTGGCTGGCTGGACTCCTC | 130 | 103.96 | NC_052543.1 | |
| *UQCRC2* | GTAGCGCCGAAAGTTGCAGT | CGCCCGGACACAGCTTCA | 58 | 91.14 | NC_052545.1 | |

**Table B:** PCR conditions used for all PCR reactions during evaluation of specificity, using Dream Taq (Thermo Fisher scientific Inc., Massachusetts, USA).

| Temperature [°C] | Time |  |
| --- | --- | --- |
| 95 | 2 min |  |
| 95 | 30 s | 30× |
| 60 | 30 s |  |
| 72 | 1 min |  |
| 72 | 5 min |  |
| 4 | ∞ |  |

**Table C:** Thermal cycling parameters used on the final qPCR runs according to the manufacturers protocol (Fluidigm Corporation, San Francisco, USA).

| Step | Flex Six | | 96×96 | |
| --- | --- | --- | --- | --- |
|  | Temperature [°C] | duration | Temperature [°C] | duration |
| Thermal mixing | 25 | 30 min | 70 | 40 min |
|  | 70 | 60 min | 60 | 30 sec |
| Hot start | 95 | 1 min | 95 | 1 min |
| PCR, 30 cycles | 96 | 5 sec | 96 | 5 sec |
|  | 60 | 20 sec | 60 | 20 sec |
| Melting curve | 60 | 3 sec | 60 | 3 sec |
|  | 60-95 | 1°C/3sec | 60-95 | 1°C/3sec |
