# Supplementary material for: The dynamics of mitochondrial-linked gene expression among tissues and life stages in two contrasting strains of laying hens
Source: PLoS One. 2022 Jan 13;17(1):e0262613. doi: 10.1371/journal.pone.0262613 (PMC8757906; doi:10.1371/journal.pone.0262613)
Supplement: S4 File — (DOCX) [file pone.0262613.s004.docx]

**Table A:** **Genes that showed a decrease in gene expression with ongoing periods.** Shown are emmeans and standard errors derived from the statistical model. Pairwise Tukey HSD tests were used to test for significance.

| gene | emmean± SE | emmean± SE | p-value |
| --- | --- | --- | --- |
|  | Period 1 | Period 5 |  |
| *ATP5F1* | 1.9±0.0941 | 1.47±0.093 | 0.0122 |
| *GAPDH* | 2.67±0.0789 | 2.34±0.0808 | 0.0254 |
| *IGF-1α* | 2.298±0.124 | 0.989±0.122 | <0.0001 |
| *MTOR* | 0.920±0.106 | 0.476±0.106 | 0.0249 |
| *PGC1α* | 2.23±0.0904 | 1.5±0.0902 | <0.0001 |
| *UQCRC1* | 1.94±0.128 | 1.49±0.129 | 0.0171 |
|  | Period 2 | Period 5 |  |
| *ATP5F1* | 1.88±0.0947 | 1.47±0.093 | 0.0182 |
| *PRKAA1* | 0.869±0.0937 | 0.562±0.0908 | 0.0085 |
|  | Period 1 | Period 4 |  |
| *PGC1α* | 2.23±0.0904 | 1.47±0.0878 | <0.0001 |

**Table B:** **Genes that showed an increase in gene expression with ongoing periods**. Shown are emmeans and standard errors derived from the statistical model. Pairwise Tukey HSD tests were used to test for significance.

| gene | emmean± SE | emmean± SE | p-value |
| --- | --- | --- | --- |
|  | Period 2 | Period 5 |  |
| *ATP6* | 0.842±0.187 | 1.78±0.186 | 0.0004 |
| *COX1* | -0.60754±0.243 | 0.56321±0.246 | 0.0015 |
| *COX3* | -0.256±0.172 | 0.794±0.173 | <0.0001 |
| *CytB* | 0.339±0.165 | 1.215±0.167 | 0.0002 |
| *ND1* | -0.026±0.188 | 1.004±0.189 | 0.0002 |
| *ND4* | -0.0557± 0.195 | 1.0259± 0.198 | 0.0003 |
| *ND4L* | 0.833±0.151 | 1.553±0.153 | 0.0073 |
| *NDUFB6* | 0.103±0-151 | 0.760±0.148 | 0.0105 |
| *SOD2* | 0.82±0.0857 | 1.23±0.086 | 0.0046 |
|  | Period 2 | Period 3 |  |
| *COX3* | -0.256±0.172 | 0.358±0.169 | 0.0329 |
| *ND1* | -0.026±0.188 | 0.673±0.185 | 0.0208 |
