# Supplementary material for: The dynamics of mitochondrial-linked gene expression among tissues and life stages in two contrasting strains of laying hens
Source: PLoS One. 2022 Jan 13;17(1):e0262613. doi: 10.1371/journal.pone.0262613 (PMC8757906; doi:10.1371/journal.pone.0262613)
Supplement: S1 Table — p-values from the three-factorial anova obtained from the linear mixed model. Statistical significance was declared when p < 0.05. (DOCX) [file pone.0262613.s006.docx]

**S1 Table:** **Significant influence of strain, period, tissue and all possible interactions on gene expression per gene.** p-values from the three-factorial anova obtained from the linear mixed model. Statistical significance was declared when p < 0.05.

| Gene | strain | period | tissue | Tissue:period | Strain:tissue | Strain:period | Strain:tissue:period |
| --- | --- | --- | --- | --- | --- | --- | --- |
| *ATP6* | ns | 0.00197 | <2.2E-16 | 0.009087 | ns | ns | ns |
| *ATP5F1* | ns | 0.007754 | <2.2E-16 | 0.005582 | ns | ns | ns |
| *ATP8* | ns | ns | <2.2E-16 | 3.76E-05 | ns | ns | ns |
| *COXC6* | ns | ns | <2.2E-16 | ns | ns | ns | ns |
| *COX1* | ns | 0.005758 | <2.2E-16 | ns | ns | ns | ns |
| *COX2* | ns | ns | <2.2E-16 | 0.01084 | ns | ns | ns |
| *COX3* | ns | 0.0002235 | <2.2E-16 | 0.0115673 | ns | ns | ns |
| *COX5A* | ns | ns | <2.2E-16 | ns | ns | ns | 0.02761 |
| *CytB* | ns | 0.0009725 | <2.2E-16 | 0.0081368 | ns | ns | ns |
| *GAPDH* | 0.03419 | 0.02207 | <2.2E-16 | 5.01E-09 | 0.01033 | ns | ns |
| *IGF-1α* | ns | 7.69E-15 | <2.2E-16 | 1.09E-15 | ns | ns | ns |
| *MTOR* | ns | 0.02647 | <2.2E-16 | 0.001926 | ns | ns | 0.012689 |
| *ND1* | ns | 0.0007885 | <2.2E-16 | 0.0206088 | ns | ns | ns |
| *ND4* | ns | 0.001269 | <2.2E-16 | ns | ns | ns | ns |
| *ND4L* | ns | 0.01734 | <2.2E-16 | 9.99E-05 | ns | ns | ns |
| *ND5* | ns | ns | <2.2E-16 | ns | ns | ns | ns |
| *ND6* | 0.0199 | ns | <2.2E-16 | ns | ns | ns | ns |
| *NDUFB6* | ns | 0.01507 | <2.2E-16 | ns | ns | 0.04275 | ns |
| *PGC1α* | 8.92E-06 | 5.94E-09 | <2.2E-16 | 1.48E-06 | 0.003393 | ns | 0.014803 |
| *PRKAA1* | ns | 0.016245 | <2.2E-16 | 0.007395 | ns | ns | 0.006349 |
| *PRKAA2* | ns | ns | <2.2E-16 | 1.22E-06 | 0.001119 | ns | ns |
| *PRKAB2* | ns | 0.042001 | <2.2E-16 | 2.68E-09 | 0.0003496 | ns | ns |
| *PRKAG2* | ns | ns | <2.2E-16 | 0.0005211 | ns | ns | ns |
| *SDHA* | ns | ns | <2.2E-16 | 0.02314 | ns | ns | ns |
| *SDHB* | ns | ns | <2.2E-16 | 1.10E-08 | ns | ns | 0.0123 |
| *SOD2* | 0.00751 | 0.012412 | <2.2E-16 | 0.003144 | ns | ns | ns |
| *UQCRC1* | ns | 0.02291 | <2.2E-16 | 6.82E-05 | ns | ns | 0.03006 |
| *UQCRC2* | ns | ns | <2.2E-16 | ns | ns | ns | 0.03389 |
