# Supplementary material for: The dynamics of mitochondrial-linked gene expression among tissues and life stages in two contrasting strains of laying hens
Source: PLoS One. 2022 Jan 13;17(1):e0262613. doi: 10.1371/journal.pone.0262613 (PMC8757906; doi:10.1371/journal.pone.0262613)
Supplement: S2 Table — (DOCX) [file pone.0262613.s007.docx]

**S2 Table. Number of samples per gene and tissue after the removal of outliers used to calculate emmeans from the statistical model.**

| Gene/tissue | Breast | Duodenum | Ileum | Liver | Ovary | Total |
| --- | --- | --- | --- | --- | --- | --- |
| ***ATP6*** | 98 | 95 | 96 | 90 | 97 | 476 |
| ***ATP8*** | 99 | 95 | 97 | 92 | 98 | 481 |
| ***COX1*** | 99 | 96 | 92 | 94 | 96 | 477 |
| ***COX2*** | 97 | 94 | 95 | 91 | 96 | 473 |
| ***COX3*** | 99 | 95 | 95 | 91 | 97 | 477 |
| ***ND1*** | 98 | 93 | 95 | 91 | 95 | 472 |
| ***ND4*** | 97 | 95 | 97 | 98 | 98 | 485 |
| ***ND4L*** | 99 | 94 | 98 | 93 | 95 | 479 |
| ***ND5*** | 75 | 60 | 65 | 53 | 43 | 296 |
| ***ND6*** | 88 | 72 | 77 | 62 | 72 | 371 |
| ***ATP5F1*** | 92 | 87 | 92 | 84 | 83 | 438 |
| ***COX5A*** | 96 | 77 | 84 | 75 | 79 | 411 |
| ***COXC6*** | 87 | 87 | 87 | 81 | 93 | 435 |
| ***CytB*** | 99 | 97 | 97 | 94 | 99 | 486 |
| ***NDUFB6*** | 86 | 87 | 86 | 81 | 93 | 433 |
| ***UQCRC1*** | 96 | 92 | 97 | 88 | 95 | 468 |
| ***UQCRC2*** | 98 | 79 | 85 | 74 | 78 | 414 |
| ***SDHA*** | 97 | 92 | 94 | 87 | 94 | 464 |
| ***SDHB*** | 93 | 96 | 98 | 94 | 97 | 478 |
| ***IGF-1*** | 96 | 88 | 91 | 87 | 90 | 452 |
| ***MTOR*** | 99 | 92 | 94 | 83 | 92 | 460 |
| ***PRKAA1*** | 93 | 86 | 95 | 83 | 90 | 447 |
| ***PRKAA2*** | 99 | 74 | 82 | 62 | 67 | 384 |
| ***PRKAB2*** | 99 | 89 | 89 | 80 | 88 | 445 |
| ***PRKAG2*** | 79 | 82 | 90 | 84 | 92 | 427 |
| ***SOD2*** | 99 | 94 | 97 | 94 | 96 | 480 |
| ***PGC1-a*** | 94 | 90 | 91 | 80 | 89 | 444 |
| ***GAPDH*** | 88 | 96 | 98 | 96 | 98 | 476 |
