# Supplementary material for: The dynamics of mitochondrial-linked gene expression among tissues and life stages in two contrasting strains of laying hens
Source: PLoS One. 2022 Jan 13;17(1):e0262613. doi: 10.1371/journal.pone.0262613 (PMC8757906; doi:10.1371/journal.pone.0262613)
Supplement: S3 Table — (DOCX) [file pone.0262613.s008.docx]

**S3 Table:** **Number of samples per gene, tissue and period after the removal of outliers used to calculate emmeans from the statistical model.**

| period | 1 | | | | | 2 | | | | | 3 | | | | |
| --- | --- | --- | --- | --- | --- | --- | --- | --- | --- | --- | --- | --- | --- | --- | --- |
| Gene/tissue | breast | duodenum | ileum | liver | ovary | breast | duodenum | ileum | liver | ovary | breast | duodenum | ileum | liver | ovary |
| ***ATP6*** | *20* | 19 | 19 | 19 | 18 | 20 | 18 | 19 | 16 | 20 | 20 | 20 | 19 | 18 | 20 |
| ***ATP8*** | *20* | 18 | 19 | 19 | 19 | 20 | 19 | 20 | 18 | 20 | 20 | 20 | 19 | 19 | 20 |
| ***COX1*** | *20* | 20 | 18 | 19 | 18 | 20 | 18 | 18 | 19 | 20 | 20 | 20 | 19 | 18 | 20 |
| ***COX2*** | *20* | 18 | 19 | 19 | 18 | 20 | 19 | 19 | 18 | 19 | 20 | 20 | 19 | 18 | 20 |
| ***COX3*** | *20* | 18 | 18 | 19 | 19 | 20 | 19 | 19 | 17 | 19 | 20 | 20 | 19 | 19 | 20 |
| ***ND1*** | *20* | 18 | 20 | 20 | 18 | 20 | 17 | 19 | 17 | 19 | 19 | 20 | 19 | 19 | 20 |
| ***ND4*** | *18* | 18 | 18 | 19 | 19 | 20 | 19 | 20 | 20 | 20 | 20 | 20 | 20 | 20 | 20 |
| ***ND4L*** | *20* | 19 | 20 | 19 | 16 | 20 | 17 | 20 | 19 | 20 | 20 | 20 | 19 | 18 | 20 |
| ***ND5*** | *16* | 8 | 15 | 6 | 7 | 16 | 10 | 12 | 12 | 9 | 15 | 16 | 14 | 13 | 11 |
| ***ND6*** | *18* | 9 | 16 | 8 | 12 | 16 | 14 | 17 | 12 | 11 | 18 | 17 | 15 | 13 | 18 |
| ***ATP5F1*** | *20* | 15 | 18 | 16 | 15 | 20 | 17 | 20 | 17 | 13 | 17 | 19 | 17 | 16 | 19 |
| ***COX5A*** | *19* | 12 | 17 | 13 | 15 | 19 | 14 | 18 | 13 | 13 | 19 | 18 | 16 | 16 | 18 |
| ***COXC6*** | *19* | 17 | 17 | 18 | 18 | 19 | 15 | 17 | 15 | 17 | 19 | 18 | 18 | 18 | 20 |
| ***CytB*** | *20* | 20 | 19 | 20 | 20 | 20 | 19 | 20 | 18 | 20 | 20 | 20 | 19 | 18 | 20 |
| ***NDUFB6*** | *19* | 17 | 18 | 17 | 17 | 19 | 14 | 16 | 15 | 18 | 16 | 18 | 17 | 18 | 20 |
| ***UQCRC1*** | *20* | 18 | 19 | 18 | 17 | 19 | 17 | 20 | 16 | 20 | 20 | 20 | 19 | 18 | 20 |
| ***UQCRC2*** | *20* | 12 | 18 | 13 | 15 | 20 | 15 | 17 | 13 | 11 | 20 | 19 | 16 | 15 | 18 |
| ***SDHA*** | *20* | 18 | 19 | 17 | 18 | 20 | 17 | 20 | 16 | 18 | 20 | 19 | 19 | 18 | 20 |
| ***SDHB*** | *20* | 20 | 20 | 19 | 18 | 20 | 18 | 20 | 18 | 20 | 18 | 20 | 19 | 20 | 20 |
| ***IGF-1*** | *20* | 14 | 18 | 17 | 17 | 19 | 17 | 20 | 17 | 16 | 20 | 20 | 18 | 18 | 20 |
| ***MTOR*** | *20* | 18 | 19 | 15 | 18 | 20 | 17 | 20 | 15 | 17 | 20 | 20 | 18 | 17 | 20 |
| ***PRKAA1*** | *19* | 14 | 19 | 14 | 17 | 19 | 17 | 20 | 17 | 15 | 18 | 19 | 18 | 17 | 20 |
| ***PRKAA2*** | *20* | 9 | 17 | 9 | 13 | 20 | 14 | 18 | 13 | 10 | 20 | 17 | 15 | 13 | 18 |
| ***PRKAB2*** | *20* | 16 | 17 | 15 | 17 | 20 | 17 | 18 | 14 | 15 | 20 | 20 | 17 | 17 | 20 |
| ***PRKAG2*** | *15* | 15 | 17 | 17 | 18 | 17 | 15 | 18 | 14 | 17 | 16 | 16 | 18 | 18 | 20 |
| ***SOD2*** | *20* | 19 | 20 | 20 | 18 | 20 | 17 | 20 | 18 | 19 | 20 | 20 | 19 | 19 | 20 |
| ***PGC1-a*** | *19* | 17 | 19 | 15 | 17 | 19 | 17 | 18 | 15 | 15 | 20 | 19 | 18 | 17 | 20 |
| ***GAPDH*** | *16* | 20 | 20 | 20 | 19 | 20 | 18 | 20 | 19 | 20 | 19 | 20 | 19 | 19 | 20 |

| period | 4 | | | | | 5 | | | | |
| --- | --- | --- | --- | --- | --- | --- | --- | --- | --- | --- |
| tissue/gene | breast | duodenum | ileum | liver | ovary | breast | duodenum | ileum | liver | ovary |
| **ATP6** | 19 | 19 | 20 | 18 | 20 | 19 | 19 | 19 | 19 | 19 |
| **ATP8** | 20 | 19 | 20 | 18 | 20 | 19 | 19 | 19 | 18 | 19 |
| **COX1** | 20 | 19 | 20 | 19 | 19 | 19 | 19 | 17 | 19 | 19 |
| **COX2** | 19 | 18 | 20 | 18 | 20 | 18 | 19 | 18 | 18 | 19 |
| **COX3** | 20 | 19 | 20 | 18 | 20 | 19 | 19 | 19 | 18 | 19 |
| **ND1** | 20 | 19 | 19 | 18 | 19 | 19 | 19 | 18 | 17 | 19 |
| **ND4** | 20 | 19 | 20 | 20 | 20 | 19 | 19 | 19 | 19 | 19 |
| **ND4L** | 20 | 19 | 20 | 18 | 20 | 19 | 19 | 19 | 19 | 19 |
| **ND5** | 14 | 13 | 9 | 10 | 8 | 14 | 13 | 15 | 12 | 8 |
| **ND6** | 18 | 16 | 14 | 16 | 16 | 18 | 16 | 15 | 13 | 15 |
| **ATP5F1** | 17 | 19 | 19 | 18 | 19 | 18 | 17 | 18 | 17 | 17 |
| **COX5A** | 20 | 16 | 16 | 16 | 16 | 19 | 17 | 17 | 17 | 17 |
| **COXC6** | 14 | 18 | 18 | 14 | 19 | 16 | 19 | 17 | 16 | 19 |
| **CytB** | 20 | 19 | 20 | 19 | 20 | 19 | 19 | 19 | 19 | 19 |
| **NDUFB6** | 14 | 19 | 18 | 15 | 19 | 18 | 19 | 17 | 16 | 19 |
| **UQCRC1** | 19 | 19 | 20 | 18 | 19 | 18 | 18 | 19 | 18 | 19 |
| **UQCRC2** | 19 | 16 | 17 | 17 | 17 | 19 | 17 | 17 | 16 | 17 |
| **SDHA** | 19 | 19 | 18 | 18 | 19 | 18 | 19 | 18 | 18 | 19 |
| **SDHB** | 17 | 19 | 20 | 18 | 20 | 18 | 19 | 19 | 19 | 19 |
| **IGF-1** | 20 | 19 | 18 | 18 | 18 | 17 | 18 | 17 | 17 | 19 |
| **MTOR** | 20 | 19 | 19 | 18 | 19 | 19 | 18 | 18 | 18 | 18 |
| **PRKAA1** | 19 | 19 | 20 | 18 | 20 | 18 | 17 | 18 | 17 | 18 |
| **PRKAA2** | 20 | 17 | 17 | 12 | 12 | 19 | 17 | 15 | 15 | 14 |
| **PRKAB2** | 20 | 19 | 18 | 17 | 18 | 19 | 17 | 19 | 17 | 18 |
| **PRKAG2** | 14 | 19 | 18 | 17 | 18 | 17 | 17 | 19 | 18 | 19 |
| **SOD2** | 20 | 19 | 19 | 18 | 20 | 19 | 19 | 19 | 19 | 19 |
| **PGC1-a** | 18 | 19 | 18 | 17 | 19 | 18 | 18 | 18 | 16 | 18 |
| **GAPDH** | 18 | 19 | 20 | 19 | 20 | 15 | 19 | 19 | 19 | 19 |
