# Supplementary material for: The dynamics of mitochondrial-linked gene expression among tissues and life stages in two contrasting strains of laying hens
Source: PLoS One. 2022 Jan 13;17(1):e0262613. doi: 10.1371/journal.pone.0262613 (PMC8757906; doi:10.1371/journal.pone.0262613)
Supplement: S4 Table — Included are only genes influenced by period shown in Fig 4. (DOCX) [file pone.0262613.s009.docx]

**S4 Table:** **Number of samples per gene and period after the removal of outliers used to calculate emmeans from the statistical model.** Included are only genes influenced by period shown in Fig 4.

| Gene | Period 1 | Period 2 | Period 3 | Period 4 | Period 5 |
| --- | --- | --- | --- | --- | --- |
| *ATP6* | 95 | 93 | 97 | 96 | 95 |
| *COX1* | 95 | 95 | 97 | 97 | 93 |
| *COX3* | 94 | 94 | 98 | 97 | 94 |
| *ND1* | 96 | 92 | 97 | 95 | 92 |
| *ND4* | 92 | 99 | 100 | 99 | 95 |
| *ND4L* | 94 | 96 | 97 | 97 | 95 |
| *ATP5F1* | 84 | 87 | 88 | 92 | 87 |
| *CytB* | 99 | 97 | 97 | 98 | 95 |
| *NDUFB6* | 88 | 82 | 89 | 85 | 89 |
| *UQCRC1* | 92 | 92 | 97 | 95 | 92 |
| *IGF-1* | 86 | 89 | 96 | 93 | 88 |
| *MTOR* | 90 | 89 | 95 | 95 | 91 |
| *PRKAA1* | 83 | 88 | 92 | 96 | 88 |
| *PRKAB2* | 85 | 84 | 94 | 92 | 90 |
| *SOD2* | 97 | 94 | 98 | 96 | 95 |
| *PGC1-a* | 87 | 84 | 94 | 91 | 88 |
| *GAPDH* | 95 | 97 | 97 | 96 | 91 |
