# Supplementary figures and images for: The dynamics of mitochondrial-linked gene expression among tissues and life stages in two contrasting strains of laying hens
Source: PLoS One. 2022 Jan 13;17(1):e0262613. doi: 10.1371/journal.pone.0262613 (PMC8757906; doi:10.1371/journal.pone.0262613)

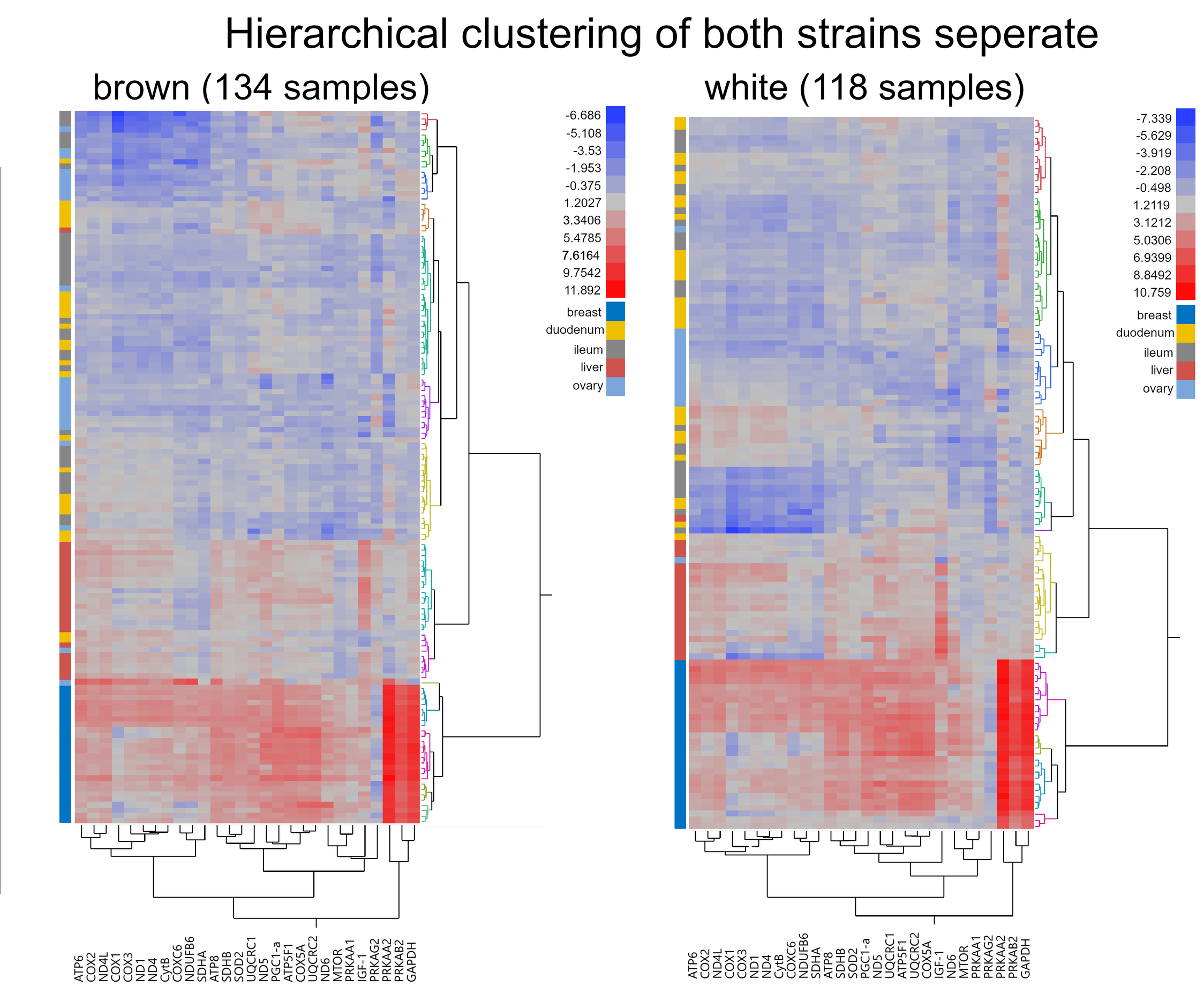

Supplement: S1 Fig — Ward’s minimum variance method [41] was used, the number clusters was estimated using the cubic clustering criterion [42]. (TIF) [file pone.0262613.s010.tif]
